# Winning the battle and losing the war? Where public health is getting it wrong in the current fight against HIV-AIDS and tuberculosis in Sub-Saharan Africa

**DOI:** 10.11604/pamj.2015.21.75.7057

**Published:** 2015-05-29

**Authors:** Luchuo Engelbert Bain, Nkeh Charles Clovis

**Affiliations:** 1 Department of Military Health, Ministry of Defense, Cameroon; 2 Centre for Population Studies and Health Promotion, CPSHP, Yaoundé, Cameroon; 3 Faculty of Medicine and Biomedical Sciences, University of Yaoundé I, Cameroon

**Keywords:** HIV, AIDS, TB, Sub-Saharan Africa

## Abstract

Despite the enormous victory that has been recorded in decreasing significantly HIV-TB related mortality and morbidity in Sub-Saharan Africa, enormous challenges continue to obstruct proper and enviable control of these conditions. These range from prioritization, ethics, funding, drug resistance and research gaps. Resistance to these diseases could constitute key treatment and prevention challenges to health care systems and the international community if not handled appropriately and promptly. This paper presents key shortcomings of the current funding, management and research policies and highlights possible action areas to remedy this situation.

## Commentary

Highly Active Anti –Retroviral Therapy (HAART) and Anti-Mycobacterium Tuberculosis (TB) therapies today have remarkably reduced HIV-TB associated morbidity and mortality. These successes however might be compromised or short lived with the growing resistant strains to available drugs. Not only health systems risk becoming unable to afford for second and third line therapies for the ever-growing affected patients with such strains, new patients risk getting infected with resistant strains [1–4]. Remarkable successes have been made to increase availability of screening tests for both conditions and drugs have been universally made available and affordable. Empirical research findings are disappointing from an ethical point of view with numerous incidence of coercion, testing without obtaining consent from parties, screened persons not aware they have the opportunity to turn down the test if they want and many clients retrospectively saying their choices were purely influenced by health care staff [5, 6]. Directive counseling and conditioning clients to get screened as a precondition to receive health care has been reported [5]. This is not only unethical, but could be counterproductive when these clients come to know they had the right to turn down these tests. Consequences could range from lack of trust in the health care system [7], that could affect not only HIV-TB care, but also other medical conditions. Participation in future research, a key corner stone to inform health policy could also be seriously compromised.

However, getting a diagnosis is no guarantee of an appropriate behavioral change later or being compliant to the prescribed therapy. Resistance to HAART and Anti-TB drugs is becoming a serious public health threat for health systems to sustain [1–4]. Second line and third line therapies are relatively expensive, scarce and at times not very effective is envisaged. As the scientific community enviably awaits complete curative therapies or vaccines for these conditions, it might be more reasonable to preserve the effectiveness of existent proven effective therapies. Making HIV testing available without taking into consideration the conditions under which this test is done is a key shortcoming of current practice. The quality of counseling and psychosocial support remain key in the management of such stigmatizing conditions like HIV and TB. The number of clinical psychologists, social workers and trained counselors in the health sector remains almost nonexistent in most health care settings in Sub-Saharan Africa. Though the level of HIV associated stigma has reduced remarkably in most countries, there is still a long way to go in most developing countries. Improving on the quality of pre and post - test counseling, coupled with psychosocial support could be the only gateways to safeguard compliance to prescribed biomedical regimes. Research funding in understanding non – compliance to effective treatment regimens has been grossly insufficient.

As the scientific community enviably awaits an HIV and TB vaccine or cure, safe - guarding effectiveness of available proven treatment options could be more realistic and cost saving. We argue that the emphasis on training and employment of clinical psychologists, counselors and social workers has been insufficient and neglected in most Sub-Saharan African Settings. HIV and TB still remain stigmatizing conditions in these settings and such actors remain unavoidable in case improved compliance is envisaged. Increasing research funding in understanding non-compliance to current biomedical regimes from a social, culture specific and sensitive perspective are compelling. Without disregarding the utmost importance of HIV-TB cure and vaccine research, we argue that it might be too early to underrate preserving effectiveness of current proven therapies. The danger could be an epidemic of highly resistant HIV-TB strains that could surpass the capacities of health care systems to contain in the years to come.
